# The Society of Academic Urology: Match Innovations and Quality Improvement

**DOI:** 10.1007/s11934-026-01378-5

**Published:** 2026-11-07

**Authors:** Modestus C. Nwogu, Danni Feng, Kirsten Greene, Lee Richstone, Simone Thavaseelan

**Affiliations:** 1 Department of Urology, Lenox Hill Hospital, New York, NY USA; 2 Department of Surgery, Division of Urology, University of Virginia Health System, Charlottesville, VA USA; 3 Division of Urology, Brown University/Rhode Island Hospital, Providence, RI USA

**Keywords:** Urology match, Quality improvement, Match innovations, Match outcomes

## Abstract

**Purpose of Review:**

The Urology Match has become increasingly competitive over the past decade, driving rising application numbers, interview inefficiencies, and growing financial and logistical burdens for both applicants and residency programs. The Society of Academic Urology (SAU) provides governance and administrative oversight of the Urology Match and in response, has implemented several quality improvements aimed at increasing transparency, efficiency, and equity across the match process. This manuscript summarizes these initiatives, and their impact on urology residency recruitment.

**Recent Findings:**

Collectively major initiatives outlined in this manuscript have improved interview distribution, reduced unnecessary application burden, enhanced communication between applicants and programs, and provided data-driven insights to guide future policy changes.

**Summary:**

As the residency application landscape continues to evolve, ongoing evaluation of these initiatives and continued collaboration among applicants, residency programs, and national organizations will be essential to further refine the match process and increase transparency and effectiveness for both applicants and residency programs.

**Supplementary Information:**

The online version contains supplementary material available at https://doi.org/10.1007/s11934-026-01378-5.

## Introduction

The Urology Match has become increasingly competitive over the past decade, with growing application volumes and a limited number of residency positions contributing to challenges in the recruitment process [1] As applicants looked to maximize their chances of matching, over-applying led to increased costs, interview hoarding, and greater administrative burden for both applicants and residency programs. These challenges highlighted inefficiencies within the residency selection process and underscored the need for continued improvement [2].

The Society of Academic Urologists (SAU) has played a leading role in addressing these challenges. As the principal organization dedicated to academic urology and residency education, the SAU works alongside national organizations, including the American Urological Association (AUA), the Association of American Medical Colleges (AAMC), the Accreditation Council for Graduate Medical Education (ACGME) and the American Board of Urology (ABU), to improve residency recruitment, medical education, and workforce development. Through its leadership, data-driven decision-making, and continuous stakeholder engagement, the SAU has introduced several innovations that have reshaped the modern Urology Match [3].

**Fig. 1:**
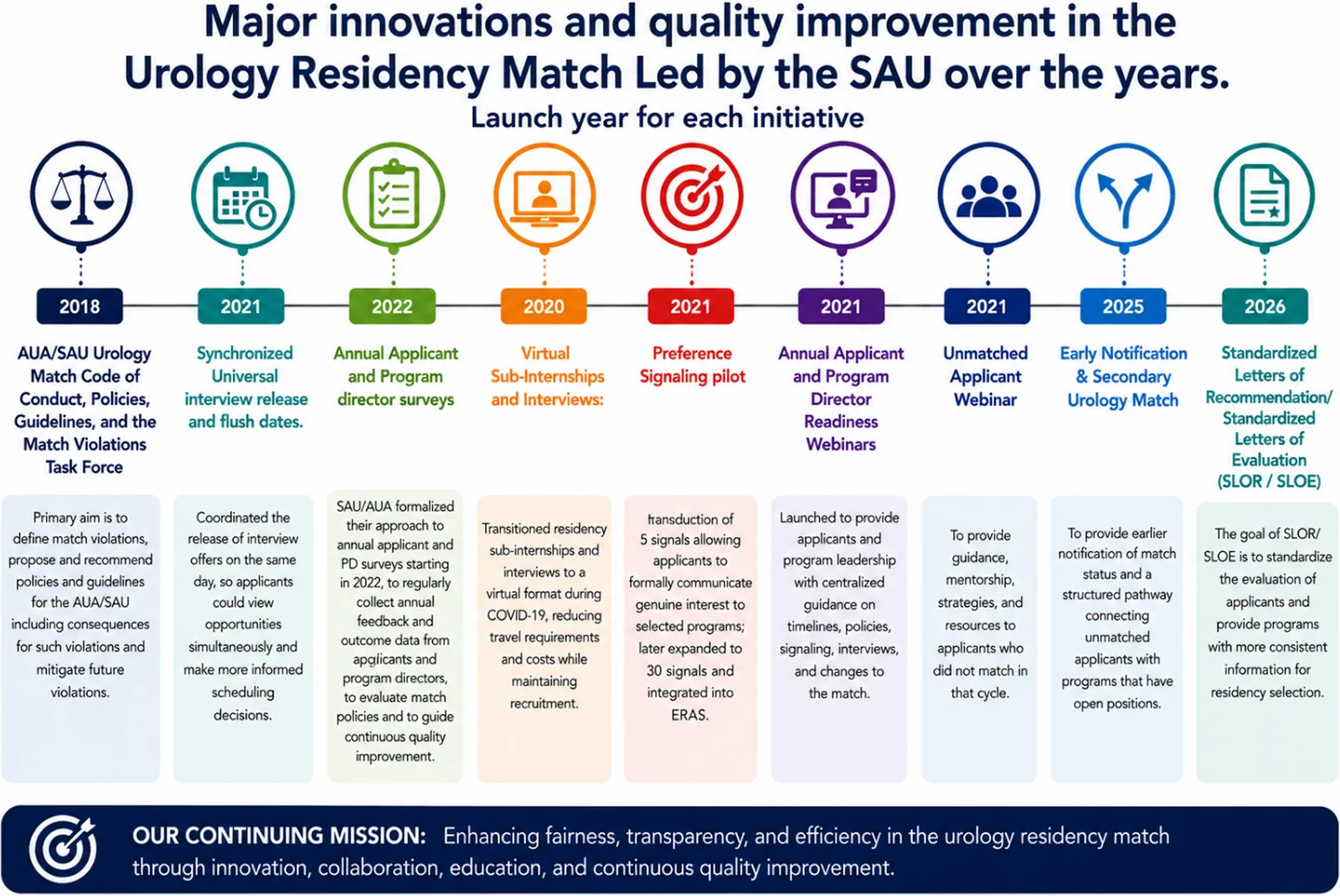
Major innovations and quality improvement in the urology residency match led by the SAU over the years

This review summarizes the major innovations introduced by the SAU over the years to improve the residency match process, and discusses the rationale behind these initiatives, the evidence supporting their implementation, their impact on applicants, residency programs, and the future direction of residency recruitment in Urology (Fig. 1).

### AUA/SAU Urology Match Code of Conduct, Policies, Guidelines, and the Match Violations Task Force

The increase in the number of applicants and changes in recruitment practices created new challenges related to communication, professionalism, and adherence to match policies. Variability in interview practices, post-interview communication, and recruitment conduct highlighted the need for standardized expectations to ensure a fair match process. To address these challenges, the SAU developed the Match Violation Task Force to review reports of potential match violations and provide recommendations on oversight to help maintain the integrity of the match [4].

The Match Violations Task Force proposed and recommended guidelines and match policies to the SAU to create a more transparent and efficient match process. These Match Guidelines were developed by the SAU based on the task force’s examination of the example of the National Resident Matching Program (NRMP) code of conduct and customized to the urology subspecialty, outlining expectations for applicants and residency programs throughout the recruitment process. These guidelines established expectations for ethical interview practices to maintain applicant privacy and provide bias free interview process and post-interview communication. It also prohibits programs from initiating contact or influencing applicants after the interview day/ranking practices and encourage professional conduct while promoting transparency and consistency across participating programs [4].

Finally for implementation, the SAU established a Match Violation Committee, that is now part of the Match Committee, as well as a reporting form on its website to investigate and adjudicate concerns over Urology Match conduct.

### Synchronized Universal Interview Release and Flush Date

Historically, interview invitations for the urology residency match were released on a rolling basis, with programs sending invitations at various times throughout the interview season. This process created prolonged uncertainty and vigilance for applicants awaiting offers, encouraged interview hoarding, and often resulted in late cancellations, limiting opportunities for other qualified applicants. In addition, applicants often felt pressured to accept interview invitations immediately, regardless of their level of interest, out of concern that declining an interview could reduce their chances of matching.

To address these challenges, the SAU introduced a coordinated universal interview release day system, followed by scheduled interview release and rebook or “Flush Day” [5]. Based on prior recruitment reform in OB Gyn and Orthopedic Surgery, under this model, participating residency programs release interview invitations on a standardized date, allowing applicants time to review and respond with all offers, waitlist and declination being disclosed before more interview offers are redistributed during subsequent flushes [6]. This coordinated approach was developed through collaboration between the SAU, residency program directors, and other national stakeholders to improve the fairness and efficiency of the interview process.

Implementation of this initiative has been associated with several benefits, including a more orderly distribution of interview opportunities, reduced applicant anxiety and vigilance surrounding interview invitations, and improved scheduling efficiency for both applicants and residency programs. The structured coordinated universal release process also requires earlier cancellation of unwanted interviews only on Flush day, allowing programs to reallocate interview slots to other applicants on time. Applicants are not permitted to cancel interviews after Flush Day, which is an effort to prevent interview hoarding and promote rescheduling to truly interested applicants. Despite these improvements, challenges such as interview hoarding, unequal interview distribution, and applicant uncertainty have not been completely eliminated.

### Annual Applicant and Program Director Surveys

To better understand the evolving landscape of the urology residency match, the SAU implemented structured annual surveys for residency applicants and PDs. These surveys were designed to identify challenges within the match process and provide data to guide evidence-based initiatives. By collecting feedback directly from both applicants and programs for each match cycle, the SAU has been able to assess the impact of these new initiatives and find areas requiring further refinement [7–10].

The applicant and PD surveys have been ongoing for years but were revised and distributed annually to all urology residency applicants at the time of rank order list submission and to PDs following the completion of the match in 2022. Survey topics have also expanded over time to include applicant demographics, research productivity, application behavior, interview experiences, preference signaling, visiting sub-internships, interview format preferences, financial considerations, and perceptions of recent match innovations [7, 8]. PD surveys similarly assess recruitment strategies, applicant evaluation methods, interview practices, and opinions on ongoing policy changes [9, 10]. Both surveys are administered consistently each cycle and their longitudinal stakeholder data have allowed meaningful year-to-year comparisons.

Findings from these surveys have directly influenced several SAU-led initiatives, including allowing programs to decide in person or virtual interview format, modifications to preference signaling, interview scheduling practices, and applicant education efforts. Survey data have shown trends in application inflation and decline, interview distribution, applicant preferences, and program recruitment practices while offering valuable insight into how recent innovations have affected both applicants and residency programs. These findings have also generated multiple peer-reviewed publications and have become a valuable resource for informing national discussions surrounding residency recruitment.

### COVID-19–Driven Virtualization of the Urology Match: Virtual Sub-Internships and Interviews

The COVID-19 pandemic significantly disrupted traditional urology residency recruitment, including visiting sub-internships and in-person interviews. In response to restrictions on away rotations, the SAU established a national working group that developed a standardized curriculum for virtual sub-internships to preserve educational opportunities and facilitate interaction between applicants and residency programs. The SAU provided a structured framework based on core educational competencies and supported implementation through national webinars and shared resources [11, 12]. Virtual Sub-Is incorporated experiences such as didactic conferences, case-based discussions, faculty and resident interactions, and, at some institutions, virtual participation in clinics and live surgical cases [11]. Margolin et al. reported that 77% of participating students and 78% of PDs rated virtual sub-internships as excellent or very good [12]. Although developed as an emergency response to the pandemic, the initiative demonstrated the feasibility of delivering components of the traditional visiting rotation through a virtual platform and provided an important bridge for applicants during a period of significant disruption to the urology match. Nevertheless, in person training could not be replicated and virtual SubIs were limited to the pandemic induced travel restrictions.

Similarly, the COVID-19 pandemic led to the rapid adoption of virtual interviews for the urology residency match, replacing the traditional in-person interview format, Table 1. While initially implemented to minimize health risks, virtual interviews also addressed longstanding concerns about the financial and logistical burden of the residency match process [13]. A survey done by the AAMC found that interviewing students spent anywhere from $400 to $7000 on travel to interviews with a median cost of $3000 [14]. By eliminating travel expenses and increasing accessibility, virtual interviews allowed applicants to interview at more programs regardless of geographic location or financial resources. Virtual interviews also promoted greater equity by reducing financial barriers that may have previously limited interview opportunities for some applicants [14].

**Table 1:** Interview format preferences in the urology residency match

Match Cycle	Interview Format	Key Findings
2020–2021	Virtual Only	Virtual Interviews were adopted during the Covid-19 pandemic to reduce travel and maintain recruitment.
2021–2022	Virtual Only	Applicants appreciated lower costs and increased accessibility; concerns remained regarding assessment of program culture and fit.
2022–2024	Predominantly Virtual	Ongoing discussions regarding return to in-person
2024–2025	In-person, Virtual and Hybrid	Most applicants preferred in-person interviews.
Future Direction	Hybrid Recruitment	Continues evaluation of hybrid interview model to balance applicant accessibility, cost, and accurate assessment of program fit.

Despite these benefits, virtual interviews introduced new challenges. Many applicants reported difficulty assessing program culture, resident camaraderie, faculty interactions, and the overall learning environment through a virtual platform. Likewise, residency programs expressed concerns about their ability to fully evaluate applicants’ interpersonal skills and program fit. These limitations contributed to reduced confidence in ranking decisions for both applicants and programs and highlighted the importance of meaningful interpersonal interactions during the recruitment process.

As pandemic-related restrictions have eased, many programs have transitioned back to in-person interviews or adopted hybrid recruitment models that involve virtual informational sessions with in-person interviews Table 1. Ongoing applicant and program director surveys continue to evaluate interview preferences and outcomes to determine the best interview format.

### Preference Signaling

Preference signaling (PS) was introduced to address the growing challenges of application inflation, unequal interview distribution and the difficulty of assessing genuine applicant interest within the Urology Match. As applicants submit an increasing number of applications each year, residency programs found it more difficult to identify genuinely interested applicants, while applicants often struggled to distinguish themselves among an increasingly competitive pool. This imbalance contributed to interview hoarding, reduced interview availability for many qualified applicants, and increased financial and administrative burdens for both applicants and residency programs [15–18].

**Fig. 2:**
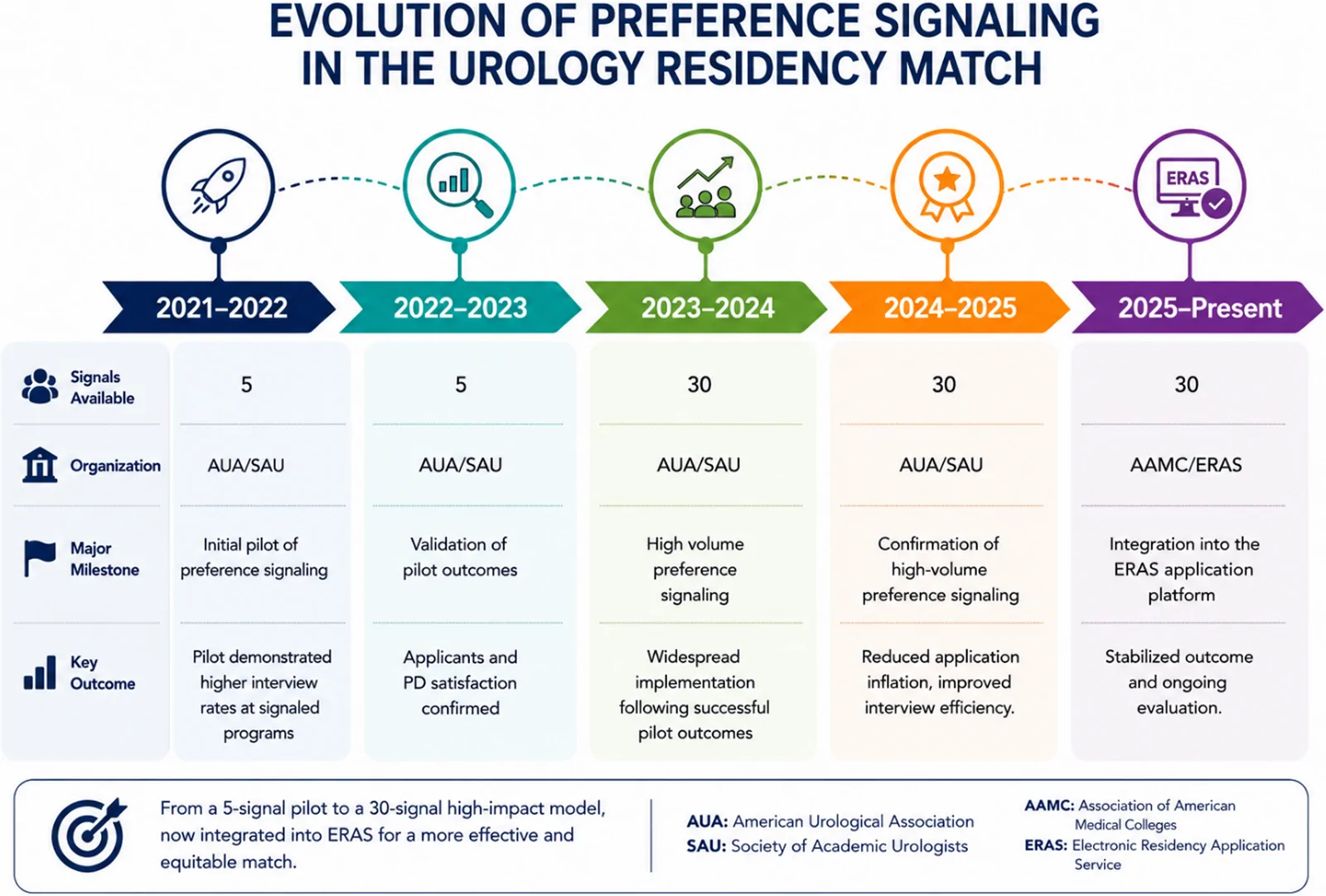
Evolution of preference signaling in the urology residency match

In response, the AUA and the SAU launched a pilot PS program during the 2021–2022 match cycle based on the prior example of a similar pilot in otolaryngology. Applicants were initially provided with 5 PS, allowing them to select residency programs of greatest interest, Fig. 2. Following encouraging early results, the number of available signals increased to 30 PS. Responsibility for administering PS later transitioned from the AUA/SAU to the Association of American Medical Colleges (AAMC) through the Electronic Residency Application Service (ERAS), allowing integration of signaling into the standardized residency application platform [16].

Multiple studies have consistently shown the effectiveness of PS in improving interview allocation. The original pilot study showed that applicants were 5 times more likely to receive interview invitations from signaled programs than from non-signaled programs [17]. Subsequently, applicant surveys and data analyses have confirmed these findings, demonstrating that PS improves interview yield while providing residency programs with a reliable indicator of applicant interest. Program directors have also reported that PS aid in holistic application review and facilitate interview selection among a large applicant pool [18].

PS has become one of the most impactful innovations in the urology residency match by allowing applicants to communicate genuine program interest without imposing formal limits on the number of applications submitted. In the 2025 urology residency match, 81% of surveyed Program directors used preference signaling as an initial application screening tool [10]. By allowing applicants to express genuine program interest, signaling has improved interview efficiency, encouraged more strategic application behavior, and provided residency programs with more information to guide interview decisions. The expansion from low volume to high volume PS has coincided with a substantial reduction in application volume while maintaining stable interview opportunities. According to the 2026 SAU Applicant Survey, the mean number of applications submitted per applicant decreased from 67.5 in 2024 to 51.8 in 2025 and 44.1 in 2026 (*P* < 0.001), while mean interview offers remained relatively unchanged at 14.2, 14.1, and 14.0, respectively [1]. Furthermore, 77.7% of respondents in 2026 SAU Applicant Survey also reported that the increased number of signals allowed them to apply to fewer programs. These findings suggest that high-volume PS may contribute to more efficient application behavior without reducing interview opportunities [1].

Despite these successes, several questions remain regarding the best implementation of PS. The ideal number of signals, the influence of geographic preferences, variability in signaling strategies among applicants, and the equitable use of signals across different applicant groups continue to be areas of active investigation. Ongoing annual applicant and program director surveys will remain essential for evaluating these issues and guiding future policy modifications.

### Annual Applicant and Program Director (PD) Readiness Webinars

To improve communication and increase transparency throughout the residency application process, SAU introduced annual applicant and PD readiness webinars. These webinars were developed to educate, advise and support applicants and residency programs on the evolving match process, clarify important timelines and policies, and provide guidance on new initiatives such as preference signaling, interview scheduling, and application strategies. By creating a centralized educational platform, the SAU sought to reduce uncertainty and promote a more standardized stakeholder education and onboarding of the match process [19].

The webinars are conducted annually before the start of each match cycle and include separate sessions for applicants and residency program directors [20]. Applicant-focused webinars typically address application preparation, preference signaling, interview expectations, and frequently asked questions regarding the match, while program director sessions discuss recruitment strategies, applicant evaluation, and implementation of new match policies. Interactive question-and-answer sessions allow participants to receive clarification directly from SAU leadership and other experts involved in residency recruitment.

Survey feedback has demonstrated that applicants value the webinars as a reliable source of information, while program directors have reported improved consistency in understanding and implementing new recruitment practices. The webinars have also offered an effective mechanism for communicating policy changes and gathering stakeholder feedback that informs future quality improvement efforts.

## Early Notification, Unmatched Applicant Webinar and the Secondary Urology Match

Historically, applicants who did not match into urology had limited time and structured guidance to pursue available positions following the primary match. Similarly, residency programs with unfilled positions, newly accredited programs, or approved complement increases lacked a standardized mechanism to identify eligible unmatched applicants [21]. Unlike the NRMP Main Residency Match, urology operates through an early match process in which applicants traditionally received their match results electronically without prior notification of their matched or unmatched status. For unmatched applicants, receiving this news on the same day others celebrated their successful matches could be particularly difficult, with limited time to process the outcome and explore alternative opportunities.

To address these challenges, the SAU in collaboration with the AUA and AAMC developed an early notification and secondary match process.

Early notification allows both matched and unmatched applicants and unfilled programs to learn their matched versus unmatched status before final and individual match results are released, giving additional time for unmatched applicants to grieve privately, seek guidance and prepare for available opportunities. Medical school leadership can also access the match status of their students, allowing advisors to provide timely support to unmatched applicants. The subsequent Secondary Urology Match provides a structured pathway for eligible applicants to pursue positions at programs with unfilled vacancies, including opportunities resulting from new programs or recently approved complement increases that miss the timeline for match registration.

In addition to these structural changes, the SAU has provided dedicated webinars for unmatched urology applicants. These sessions offer guidance, strategies, resources, and opportunities for discussion with program directors, mentors, residents, and coordinators. Unmatched applicant webinars have been offered by the SAU since at least 2021 and complement the secondary match by providing educational and mentorship support during a challenging transition period.

### Standardized Letters of Recommendation/Standardized Letters of Evaluation (SLOR/SLOE)

Letters of recommendation have long been one of the most important components of the urology residency application process. However, traditional narrative letters often varied considerably in content, structure, and quality, making it difficult for residency programs to objectively compare applicants. Differences in writing style, grading practices, and implicit bias also contributed to variability in applicant assessment, limiting the ability of programs to consistently interpret letter content [22].

**Fig. 3:**
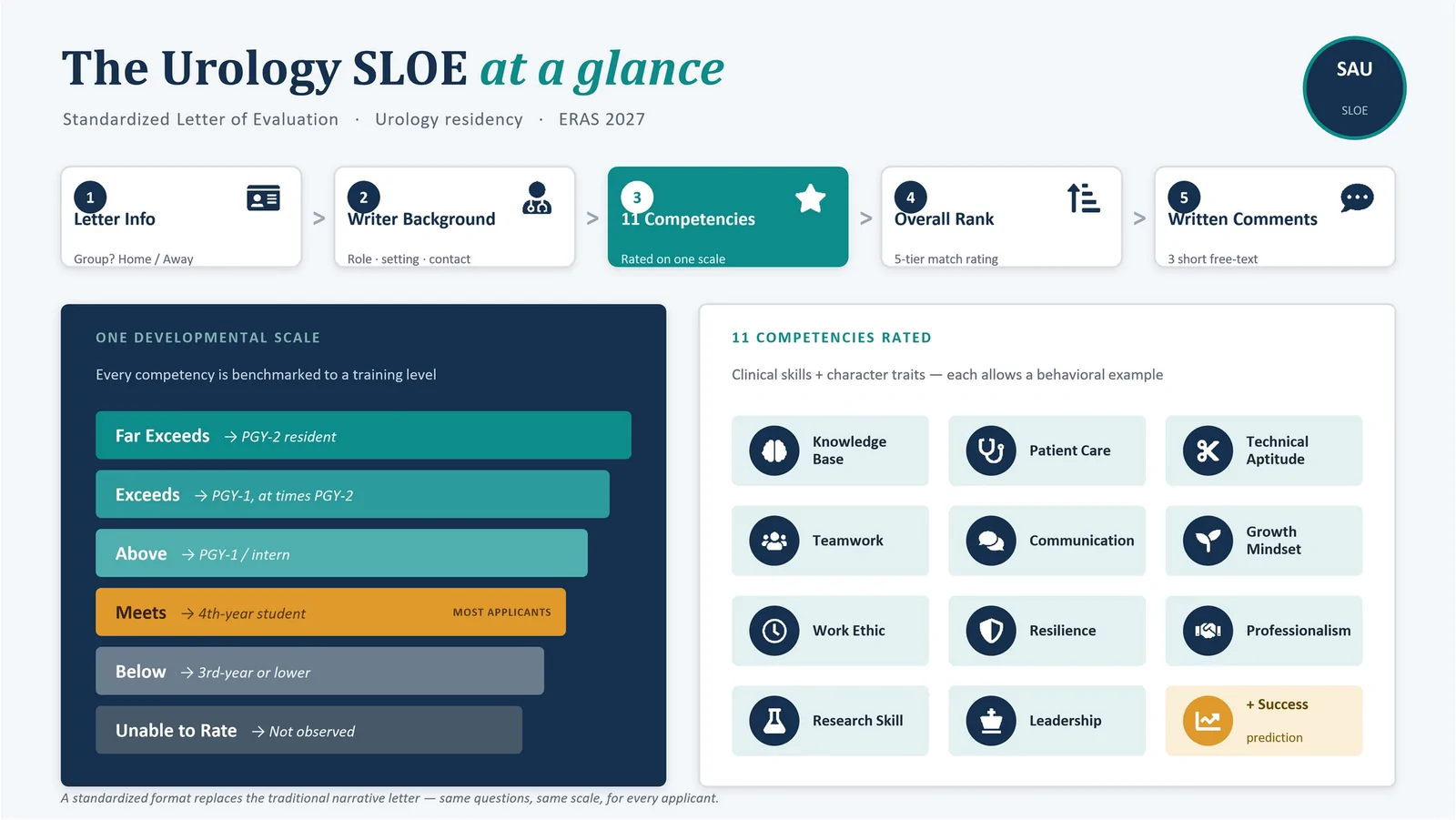
Overview of standardized letter of evaluation

To improve the consistency and objectivity of applicant evaluation, the SAU developed the Standardized Letter of Recommendation which provided a structured format for evaluating applicants across several competency domains. Building on this foundation, the SAU has recently transitioned toward the Standardized Letter of Evaluation (SLOE) Fig. 3, which incorporates a more comprehensive and standardized assessment of applicant performance while providing residency programs with clearer, more comparable information during the selection process. The current SLOE is being implemented this year 2026 and two letters in this format are required for each applicant (Supplementary Material 1).

The transition from a standard narrative letter of recommendation to SLOE is expected to improve the objectivity and consistency of residency applications by reducing variability among evaluators and promoting more standardized comparisons across applicants [23]. A structured evaluation format may also enhance holistic review by providing programs with clearer information about clinical performance, professionalism, communication skills, work ethic, and overall readiness for residency.

As implementation of the SLOE continues, ongoing evaluation will be essential to determine its impact on residency recruitment. Studies on SLOE are set to begin at the end of the 2026–2027 Match and will be assessing the reliability, validity, and acceptance of the new evaluation format among applicants, letter writers, and residency programs. Findings from these studies, along with future publications and continued collaboration among the SAU and national organizations, will help refine the SLOE and determine its long-term role in the urology residency match.

## Conclusion

The SAU has played a pivotal role in modernizing the urology residency match through innovations that have improved transparency, efficiency, and continuous quality improvement. Initiatives including preference signaling, universal interview release, annual applicant and program director surveys, virtual interviews, educational webinars, the secondary match, and the transition to the Standardized Letter of Evaluation reflect a sustained commitment to data-driven policy development and stakeholder engagement. As these initiatives continue to change, ongoing collaboration and rigorous evaluation will be essential to further strengthen the residency selection process and support the next generation of academic urologists. The continued success of the urology residency match will depend not on a single innovation, but on the SAU’s ongoing commitment to data-driven evaluation, collaboration, and continuous quality improvement.

## Key References

- Traxel, E., & Thavaseelan, S. Preference Signaling in the Urology Match. Current Urology Reports. 2024;25:267–70. https://doi.org/10.1007/s11934-024-01210-y.
  - ○ Very Important: This article written by one of the coauthors ST, summarizes the introduction, evolution, and outcomes of preference signaling in Urology Match and its use in other medical specialties. By so doing provides a framework to include its impact on the innovations and quality improvement of the Match process.
- Creswell M, Tverye A, Richstone L, Thavaseelan S, Badalato G, Sorensen MD, Greene K, Lallas C, Keel C, Faris S, Yates J, May D, Kowalik C, Mirza M. Evaluating the 2025 Urology Residency Match: An Analysis of Applicant Preference Signaling, Interview Formats, and Match Outcomes. Urology. 2026;S0090-4295(26):00524-8. https://doi.org/10.1016/j.urology.2026.08.016.
  - ○ Very Important: This article showed and analyzed how recent changes in the urology match, including interview formats and preference signals (PS), affected match outcomes positively in 2025 Urology Residency Match. These Innovations aimed at making urology more transparent and efficient, its positive effects and impact were summarized in this article.
- Pingle, S., Margolin, E., Creswell, M., Faris, S., Greene, K., Keel, C., Richstone, L., Sorensen, M. D., Thavaseelan, S., Traxel, E., Mirza, M., & Badalato, G. The 2025 Urology Match: Results From the Society of Academic Urologists Medical Student Survey. Urology. 2025. https://doi.org/10.1016/j.urology.2025.07.025.
  - ○ Important: This study looked at medical Students perspectives on the 2025 match and how these different Match Innovations and quality improvement has made Match more efficient and transparent. How preference signaling, Universal Interview release day/Flush date etc. has improved match and how applicants perceive the process.

## Supplementary Information

Below is the link to the electronic supplementary material.

Supplementary Material 1 (PDF 347 KB)

## Data Availability

No datasets were generated or analysed during the current study.
