# Supplementary material for: The Society of Academic Urology: Match Innovations and Quality Improvement
Source: Curr Urol Rep. 2026 Nov 7;27(1):62. doi: 10.1007/s11934-026-01378-5 (PMC13744509; doi:10.1007/s11934-026-01378-5)
Supplement: Supplementary file 1 [file 11934_2026_1378_MOESM1_ESM.pdf]

Urology Standardized Letter of Evaluation

This sample template outlines the questions included in the standardized letter of evaluation for a Urology residency application in the 2027 ERAS® application season. It is intended for preparation purposes only and should not be uploaded. Letter writers must complete and submit evaluations using the standardized letter platform within the AAMC Letter Writer Portal.

Additional Letter Information

1. Is this a group letter?

- ☐ Yes
- ☐ No

Additional Author Name(s) and Position(s) (Please specify)

____

2. Is this applicant a Home or Away student?

- ☐ Home—*NOTE:* Home *may be selected by an "adopted" home program where the applicant has spent significant time if an applicant attends a medical school without an affiliated urology residency program.*
- ☐ Away – *NOTE: Away should be selected if the applicant rotated in your department as external rotators ("Sub-I's".)*
- ☐ Not applicable

Writer Background

3. On average, in a given year, how many urology Sub-Is do you evaluate who apply to urology?
(dropdown options: 0, 1-9, 10-19, 20-29, 30-39, 40-49, 50-59…90-99, 100+)

____

4. What was the nature of your relationship with the applicant? (select all that apply)
I was/am their…

- ☐ Clinical faculty/supervisor (i.e., oversaw and provided hands-on guidance or feedback when applicant saw patients)
- ☐ Clerkship supervisor/director (i.e., responsible for rotation curriculum and evaluation)
- ☐ Research mentor
- ☐ Specialty/Career advisor
- ☐ Associate Residency Program Director
- ☐ Department Chair/Section Chief
- ☐ Residency Program Director (i.e., if you were the applicant's official program director while they were a resident in training)
- ☐ I did not interact directly with the applicant
- ☐ Other (Please describe):

DO NOT UPLOAD

5. In what setting(s) did you observe the applicant's performance? (select all that apply)

- ☐ Didactics, conference, or classroom
- ☐ Research (lab, research project, or writing a paper)
- ☐ Operating room/procedure clinic
- ☐ Inpatient service
- ☐ Outpatient service/clinic
- ☐ Private Practice
- ☐ Extracurricular/Volunteer/Committee activity
- ☐ Feedback from others (e.g., faculty, fellows, residents)
- ☐ Other (please describe):

6. Over what period did you interact with the applicant in this capacity?

-

7. What was your typical level of contact with the applicant in this capacity?

- ☐ Less than once a week
- ☐ Once a week
- ☐ Several times a week
- ☐ Daily

Applicant Evaluation

Evaluate the applicant's proficiency on each competency using the following rating scale. Most applicants should receive "Meets Expectations" ratings because this reflects expected performance level of an experienced 4th year medical student. If the applicant is rated in the "Far Exceeds Expectations" category, use the space provided to justify your rating.

Far Exceeds Expectations - Functions at the expected level of a PGY-2 urology resident.

Exceeds Expectations - Mostly functions at the expected level of a PGY-1/intern, but occasionally functions at the expected level of a PGY-2 urology resident.

Above Expectations - Functions at the expected level of a PGY-1/intern.

Meets Expectations - Functions at the expected level of a 4th year medical student.

Below Expectations - Functions at the expected level of a 3rd year or lower medical student.

Unable to Rate - No opportunity to observe performance.

8. **Knowledge Base** – Level of general and urologic medical knowledge.

If you selected *Far Exceeds Expectations* for **Knowledge Base**, please provide a behavioral example(s) to justify your rating. (50 words/300 characters)

DO NOT UPLOAD

9. **Patient Care** – Ability to develop and justify an appropriate differential diagnosis and a cohesive treatment plan.

If you selected *Far Exceeds Expectations* for **Patient Care**, please provide a behavioral example(s) to justify your rating. (50 words/300 characters)

10. **Technical Aptitude** – Surgical intuition, ability to learn new techniques and ability to perform basic urologic procedures.

If you selected *Far Exceeds Expectations* for **Technical Aptitude**, please provide a behavioral example(s) to justify your rating. (50 words/300 characters)

11. **Teamwork** – Engages with patients, peers, and interprofessional team members to constructively share information, support coordinated decision-making, and work effectively as part of a care team toward common goals to deliver high-quality, patient-centered care.

If you selected *Far Exceeds Expectations* for **Teamwork**, please provide a behavioral example(s) to justify your rating. (50 words/300 characters)

12. **Communication Skills** – Ability to exchange verbal, nonverbal, and written information clearly and respectfully with patients, families, and team members in diverse settings, demonstrates active listening, builds rapport, and ensures understanding to support effective interactions and shared understanding.

If you selected *Far Exceeds Expectations* for **Communication Skills**, please provide a behavioral example(s) to justify your rating. (50 words/300 characters)

DO NOT UPLOAD

13. **Growth Mindset** – Proactively identifies learning needs, formulates and implements development goals, independently seeks and integrates information to address gaps in knowledge or skills in clinical and scholarly settings, and openly receives and accepts feedback, reflects on it, and adjusts their behavior based on that feedback to improve performance and professional growth.

If you selected *Far Exceeds Expectations* for **Growth Mindset**, please provide a behavioral example(s) to justify your rating. (50 words/300 characters)

14. **Work Ethic** – Fulfills responsibilities reliably and on time, follows through on tasks, and is accountable for their actions and outcomes.

If you selected *Far Exceeds Expectations* for **Work Ethic**, please provide a behavioral example(s) to justify your rating. (50 words/300 characters)

15. **Resilience** – Perseveres and maintains composure under pressure by quickly adapting to change, adjusts behavior in response to new conditions or challenges, seeks appropriate support and resources when needed, and recovers from setbacks while balancing well-being with responsibilities.

If you selected *Far Exceeds Expectations* for **Resilience**, please provide a behavioral example(s) to justify your rating. (50 words/300 characters)

DO NOT UPLOAD

16. **Professionalism** – Acts with honesty and integrity, treats others with respect, upholds ethical standards, and adheres to workplace expectations and standards.

If you selected *Far Exceeds Expectations* for **Professionalism**, please provide a behavioral example(s) to justify your rating. (50 words/300 characters)

17. **Research Skill & Motivation** – Actively engages in research by contributing to one or more stages of a research project (e.g., literature review, data collection, analysis) and demonstrates genuine interest and commitment to conducting research and advancing academic knowledge.

If you selected *Far Exceeds Expectations* for **Research Skill & Motivation**, please provide a behavioral example(s) to justify your rating. (50 words/300 characters)

18. **Leadership Potential** – Ability to lead, influence, or guide other individuals or a team.

If you selected *Far Exceeds Expectations* for **Leadership Potential**, please provide a behavioral example(s) to justify your rating. (50 words/300 characters)

Overall Evaluation

19. What is your prediction of success for this applicant in a urology residency?

20. If you were ranking this applicant for your residency program they would be…

- ☐ Would not rank
- ☐ Lower rank (better than not matching)
- ☐ Middle rank (would be fine if they match with us)
- ☐ Higher rank (would be excited if they match with us)
- ☐ Highest Rank (top choice for matching with us)
- ☐ Not applicable

DO NOT UPLOAD

Written Comments

21. Please describe what makes this applicant stand out the most from their peers that will contribute to their success as a resident. (50 words/300 characters)

22. Please describe where this applicant can develop their skills to become a more well-rounded resident. (50 words/300 characters)

23. Please include any additional comments (not addressed above) about why you think this applicant will be well-suited to a career urology (may include comments from non-faculty, e.g., residents, staff). (100 words or less/ 800 characters)

DO NOT UPLOAD
